# 4-Formyl-2-nitro­phenyl 3-nitro-2-methyl­benzoate

**DOI:** 10.1107/S1600536813032583

**Published:** 2013-12-07

**Authors:** Rodolfo Moreno-Fuquen, Geraldine Hernández, Alan R. Kennedy

**Affiliations:** a Departamento de Química – Facultad de Ciencias, Universidad del Valle, Apartado 25360, Santiago de Cali, Colombia; b WestCHEM, Department of Pure and Applied Chemistry, University of Strathclyde, 295 Cathedral Street, Glasgow G1 1XL, Scotland

## Abstract

In the title formyl nitro aryl benzoate derivative, C_15_H_10_N_2_O_7_, the benzene rings form a dihedral angle of 4.96 (3)°. The mean plane of the central ester group, C—O—C–(=O)—C (r.m.s. deviation = 0.0484 Å), is twisted away from the formyl nitro aryl and benzoate rings by 46.61 (5) and 49.93 (5)°, respectively. In the crystal, the mol­ecules are packed forming C—H⋯O inter­actions in chains which propagate along [010]. Edge-fused *R* ^3^_3_(15) rings are generated along this direction.

## Related literature   

For similar formyl nitro aryl benzoate compounds, see: Moreno-Fuquen *et al.* (2013*a* ▶,*b* ▶). For information on hydrogen bonds, see: Nardelli (1995 ▶). For hydrogen-bond graph-sets motifs, see: Etter (1990 ▶).
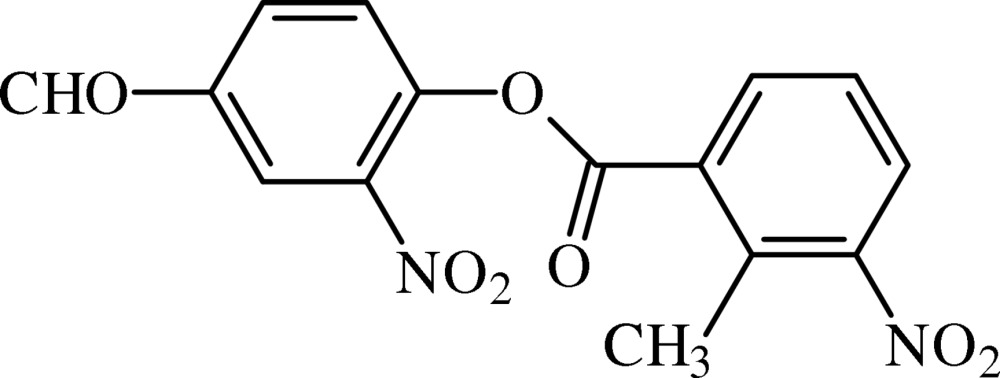


## Experimental   

### 

#### Crystal data  

C_15_H_10_N_2_O_7_
*M* *_r_* = 330.25
Monoclinic, 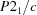

*a* = 12.7162 (5) Å
*b* = 8.0719 (2) Å
*c* = 14.1156 (5) Å
β = 110.877 (4)°
*V* = 1353.76 (8) Å^3^
*Z* = 4
Mo *K*α radiation
μ = 0.13 mm^−1^
*T* = 123 K
0.35 × 0.30 × 0.20 mm

#### Data collection  

Oxford Diffraction Xcalibur E diffractometer
6641 measured reflections
3319 independent reflections
2706 reflections with *I* > 2σ(*I*)
*R*_int_ = 0.020

#### Refinement  

*R*[*F* ^2^ > 2σ(*F* ^2^)] = 0.040
*wR*(*F* ^2^) = 0.098
*S* = 1.04
3319 reflections
222 parameters
H atoms treated by a mixture of independent and constrained refinement
Δρ_max_ = 0.32 e Å^−3^
Δρ_min_ = −0.33 e Å^−3^

### 

Data collection: *CrysAlis PRO* (Oxford Diffraction, 2010 ▶); cell refinement: *CrysAlis PRO*; data reduction: *CrysAlis PRO*; program(s) used to solve structure: *SHELXS97* (Sheldrick, 2008 ▶); program(s) used to refine structure: *SHELXL97* (Sheldrick, 2008 ▶); molecular graphics: *ORTEP-3 for Windows* (Farrugia, 2012 ▶) and *Mercury* (Macrae *et al.*, 2006 ▶); software used to prepare material for publication: *WinGX* (Farrugia, 2012 ▶).

## Supplementary Material

Crystal structure: contains datablock(s) I, global. DOI: 10.1107/S1600536813032583/ng5349sup1.cif

Structure factors: contains datablock(s) I. DOI: 10.1107/S1600536813032583/ng5349Isup2.hkl

Supporting information file. DOI: 10.1107/S1600536813032583/ng5349Isup3.cml

Additional supporting information:  crystallographic information; 3D view; checkCIF report

## Figures and Tables

**Table 1:** Hydrogen-bond geometry (Å, °)

*D*—H⋯*A*	*D*—H	H⋯*A*	*D*⋯*A*	*D*—H⋯*A*
C10—H10⋯O5^i^	0.95	2.48	3.3457 (18)	152
C12—H12⋯O4^ii^	0.95	2.71	3.5321 (19)	145

Symmetry codes: (i) 
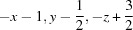
; (ii) 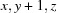.

**Fig. 1.**
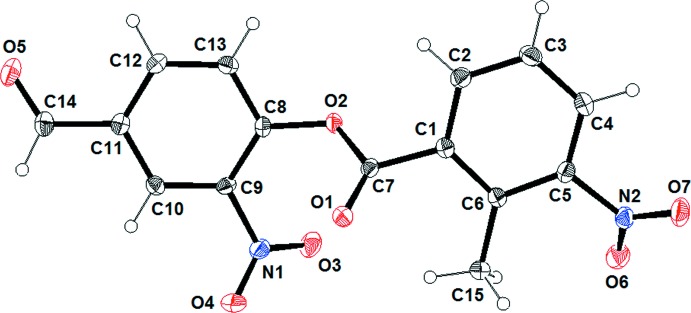
Molecular conformation and atom numbering scheme for the title compound with displacement ellipsoids drawn at the 50% probability level. H atoms are shown as spheres of arbitrary radius.

**Fig. 2.**
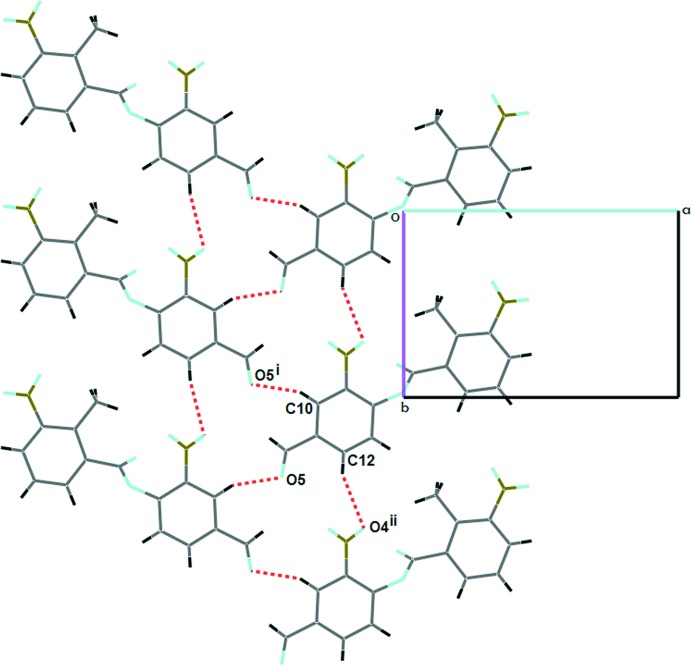
Part of the crystal structure of (I), showing the formation of helical chains which running along [010]. Symmetry code: (i) -x-1,+y-1/2,-z+1/2+1; (ii) x,+y+1,+z.

## supplementary crystallographic information

### 1. Comment

The title compound, 4-formyl-2-nitrophenyl 3-nitro-2-methyl benzoate, (I), was synthesized in order to complement the structural information on the formyl nitro aryl benzoates presented in earlier jobs from our research group: 4-formyl-2-nitrophenyl 4-bromo benzoate (F4BrB) (Moreno-Fuquen *et al.*, 2013*a*) and 4-formyl-2-nitrophenyl 4-cloro benzoate (F2ClB) (Moreno-Fuquen *et al.*, 2013*b*). The molecular structure of (I) is shown in Fig. 1. Bond lengths and bond angles show marked similarity with their homologues F4BrB and F2ClB. The benzene rings of (I) form a dihedral angle of 4.96 (3)°, a value which is quite different from the values reported for F4BrB [62.90 (7)°] and F2ClB [19.55 (9)°] similar systems. This planar arrangement may be motivated by the intermolecular interaction between the methyl group and the nitro group of the formyl ring. The ester group C8-O2-C7(O1)-C1 is planar [r.m.s. deviation= 0.0484 Å] and is twisted away from the formyl nitro aryl and benzoate rings by 46.61 (5)° and 49.93 (5)° respectively. The nitro groups form dihedral angles with the adjacent benzene rings of 37.62 (5)° for O3-N1-O4 and 39.67 (5)° for O6-N2-O7. The crystal packing shows no classical hydrogen bonds. The molecules are packed forming weak C-H···O intermolecular interactions in one-dimensional helical chains which propagates along [010] (see Fig. 2]. The C10 atom at (x,y,z) acts as hydrogen-bond donor to formyl atom O5 at (-x-1,+y-1/2,-z+1/2+1) and C12 atom at (x,y,z) acts as hydrogen-bond donor to nitro O4 atom at (x,+y+1,+z) (see Table 1; Nardelli, 1995). The combination of these two contacts generate edge-fused rings R^3^_3_(15) (Etter, 1990) along [010].

### 2. Experimental

The reagents and solvents for the synthesis were obtained from the Aldrich Chemical Co., and were used without additional purification. The title molecule was obtained through a two-step reaction. First, 3-nitro-2-methylbenzoic acid (0.502 g, 1.575 mmol) was refluxed with thionyl chloride (5 mL) in chloroform for an hour. Then, the thionyl chloride was distilled to purify the 3-nitro-2-methyl benzoyl chloride obtained as a pale-yellow translucent liquid. The same reaction flask was rearranged and an equimolar solution of 4-hydroxy-3-nitrobenzaldehyde (0.219 g, 1.575 mmol) in acetonitrile was dropped inside it with 0.03 mL of pyridine. The reaction mixture was taken to room temperature with constant stirring for about an hour. A shiny yellow solid was obtained after leaving the solvent to evaporate. IR spectra were recorded on a FT—IR SHIMADZU IR-Affinity-1 spectrophotometer. Yellow crystals; m.p 398 (1) K. IR (KBr) 3228.18cm^-1^, 3079.51 cm^-1^ (aromatic C-H); 1723.73 cm^-1^ (ester C=O); 1690.65 cm^-1^ (benzaldehyde C=O), 1264.29 cm^-1^ (ester C-O); 1568.29 cm^-1^, 1532.06 cm^-1^, 1360.66 cm^-1^, 1330.49 cm^-1^ (nitro –NO2); 1121.38 cm^-1^ (C=C).

### 3. Refinement

All H-atoms were positioned at geometrically idealized positions with C—H distance of 0.95 Å and U_iso_(H) = 1.2 times U_eq_ of the C-atoms to which they were bonded. The coordinates of the H14 atom were refined.

### Crystal data

**Table:**

C_15_H_10_N_2_O_7_	*F*(000) = 680
*M**_r_* = 330.25	*D*_x_ = 1.620 Mg m^−^^3^
Monoclinic, *P*2_1_/*c*	Melting point: 398(1) K
Hall symbol: -P 2ybc	Mo *K*α radiation, λ = 0.71073 Å
*a* = 12.7162 (5) Å	Cell parameters from 6641 reflections
*b* = 8.0719 (2) Å	θ = 3.0–29.5°
*c* = 14.1156 (5) Å	µ = 0.13 mm^−^^1^
β = 110.877 (4)°	*T* = 123 K
*V* = 1353.76 (8) Å^3^	Block, pale-yellow
*Z* = 4	0.35 × 0.30 × 0.20 mm

### Data collection

**Table:**

Oxford Diffraction Xcalibur E diffractometer	2706 reflections with *I* > 2σ(*I*)
Radiation source: fine-focus sealed tube	*R*_int_ = 0.020
Graphite monochromator	θ_max_ = 29.5°, θ_min_ = 3.0°
ω scans	*h* = −17→11
6641 measured reflections	*k* = −11→10
3319 independent reflections	*l* = −17→19

### Refinement

**Table:**

Refinement on *F*^2^	Primary atom site location: structure-invariant direct methods
Least-squares matrix: full	Secondary atom site location: difference Fourier map
*R*[*F*^2^ > 2σ(*F*^2^)] = 0.040	Hydrogen site location: inferred from neighbouring sites
*wR*(*F*^2^) = 0.098	H atoms treated by a mixture of independent and constrained refinement
*S* = 1.04	*w* = 1/[σ^2^(*F*_o_^2^) + (0.0356*P*)^2^ + 0.5883*P*] where *P* = (*F*_o_^2^ + 2*F*_c_^2^)/3
3319 reflections	(Δ/σ)_max_ < 0.001
222 parameters	Δρ_max_ = 0.32 e Å^−^^3^
0 restraints	Δρ_min_ = −0.33 e Å^−^^3^

### Special details

**Table:**

Geometry. All esds (except the esd in the dihedral angle between two l.s. planes) are estimated using the full covariance matrix. The cell esds are taken into account individually in the estimation of esds in distances, angles and torsion angles; correlations between esds in cell parameters are only used when they are defined by crystal symmetry. An approximate (isotropic) treatment of cell esds is used for estimating esds involving l.s. planes.
Refinement. Refinement of *F*^2^ against ALL reflections. The weighted *R*-factor *wR* and goodness of fit *S* are based on *F*^2^, conventional *R*-factors *R* are based on *F*, with *F* set to zero for negative *F*^2^. The threshold expression of *F*^2^ > σ(*F*^2^) is used only for calculating *R*-factors(gt) *etc*. and is not relevant to the choice of reflections for refinement. *R*-factors based on *F*^2^ are statistically about twice as large as those based on *F*, and *R*- factors based on ALL data will be even larger.

### Fractional atomic coordinates and isotropic or equivalent isotropic displacement parameters (Å^2^)

**Table:**

	*x*	*y*	*z*	*U*_iso_*/*U*_eq_	
O1	−0.02565 (8)	0.82147 (13)	0.96445 (8)	0.0165 (2)	
O2	−0.00317 (8)	0.97501 (13)	0.83848 (7)	0.0149 (2)	
O3	−0.14515 (10)	0.70025 (14)	0.76210 (9)	0.0245 (3)	
O4	−0.27370 (10)	0.70420 (14)	0.83088 (10)	0.0283 (3)	
O5	−0.44451 (10)	1.42910 (14)	0.70371 (9)	0.0253 (3)	
O6	0.32188 (10)	0.37636 (14)	0.95119 (9)	0.0256 (3)	
O7	0.45585 (9)	0.47549 (15)	1.08175 (8)	0.0246 (3)	
N1	−0.20884 (10)	0.77299 (16)	0.79668 (10)	0.0187 (3)	
N2	0.36621 (10)	0.48942 (16)	1.01005 (9)	0.0176 (3)	
C1	0.15564 (12)	0.83319 (18)	0.94637 (10)	0.0137 (3)	
C2	0.22771 (12)	0.96879 (18)	0.95875 (11)	0.0164 (3)	
H2	0.1973	1.0774	0.9445	0.020*	
C3	0.34317 (13)	0.94602 (19)	0.99163 (11)	0.0186 (3)	
H3	0.3921	1.0386	1.0017	0.022*	
C4	0.38658 (12)	0.78678 (19)	1.00971 (11)	0.0178 (3)	
H4	0.4656	0.7689	1.0333	0.021*	
C5	0.31330 (12)	0.65466 (18)	0.99296 (11)	0.0150 (3)	
C6	0.19555 (12)	0.66950 (18)	0.96223 (10)	0.0136 (3)	
C7	0.03361 (12)	0.87005 (17)	0.92016 (10)	0.0136 (3)	
C8	−0.11021 (12)	1.04391 (18)	0.81185 (10)	0.0131 (3)	
C9	−0.20853 (12)	0.95402 (18)	0.79489 (10)	0.0142 (3)	
C10	−0.31127 (12)	1.03321 (18)	0.77283 (10)	0.0149 (3)	
H10	−0.3771	0.9713	0.7660	0.018*	
C11	−0.31668 (12)	1.20438 (18)	0.76078 (10)	0.0149 (3)	
C12	−0.21895 (12)	1.29445 (18)	0.77370 (11)	0.0159 (3)	
H12	−0.2231	1.4110	0.7638	0.019*	
C13	−0.11619 (12)	1.21503 (18)	0.80080 (11)	0.0154 (3)	
H13	−0.0495	1.2775	0.8119	0.018*	
C14	−0.42791 (13)	1.2872 (2)	0.73352 (11)	0.0182 (3)	
H14	−0.4883 (14)	1.218 (2)	0.7426 (12)	0.016 (4)*	
C15	0.11801 (13)	0.52338 (19)	0.94709 (12)	0.0192 (3)	
H15A	0.0404	0.5625	0.9296	0.029*	
H15B	0.1396	0.4583	1.0097	0.029*	
H15C	0.1234	0.4540	0.8920	0.029*	

### Atomic displacement parameters (Å^2^)

**Table:**

	*U*^11^	*U*^22^	*U*^33^	*U*^12^	*U*^13^	*U*^23^
O1	0.0153 (5)	0.0170 (5)	0.0176 (5)	0.0003 (4)	0.0063 (4)	0.0016 (4)
O2	0.0115 (5)	0.0170 (5)	0.0163 (5)	0.0026 (4)	0.0049 (4)	0.0043 (4)
O3	0.0241 (6)	0.0169 (6)	0.0302 (6)	0.0036 (5)	0.0069 (5)	−0.0063 (5)
O4	0.0231 (6)	0.0180 (6)	0.0434 (7)	−0.0044 (5)	0.0113 (5)	0.0059 (5)
O5	0.0229 (6)	0.0217 (6)	0.0284 (6)	0.0090 (5)	0.0058 (5)	0.0012 (5)
O6	0.0252 (6)	0.0184 (6)	0.0321 (6)	0.0025 (5)	0.0090 (5)	−0.0027 (5)
O7	0.0176 (6)	0.0309 (7)	0.0229 (6)	0.0082 (5)	0.0041 (5)	0.0080 (5)
N1	0.0156 (6)	0.0138 (6)	0.0220 (7)	−0.0015 (5)	0.0009 (5)	−0.0005 (5)
N2	0.0155 (6)	0.0202 (7)	0.0188 (6)	0.0041 (5)	0.0082 (5)	0.0040 (5)
C1	0.0130 (7)	0.0165 (7)	0.0119 (6)	0.0006 (6)	0.0048 (5)	−0.0002 (5)
C2	0.0177 (7)	0.0148 (7)	0.0172 (7)	0.0006 (6)	0.0070 (6)	0.0001 (6)
C3	0.0160 (7)	0.0184 (8)	0.0210 (7)	−0.0037 (6)	0.0063 (6)	−0.0015 (6)
C4	0.0124 (7)	0.0224 (8)	0.0186 (7)	0.0005 (6)	0.0054 (6)	−0.0004 (6)
C5	0.0153 (7)	0.0163 (7)	0.0136 (7)	0.0037 (6)	0.0055 (5)	0.0022 (6)
C6	0.0127 (7)	0.0164 (7)	0.0118 (6)	0.0000 (6)	0.0045 (5)	0.0004 (5)
C7	0.0132 (7)	0.0115 (7)	0.0145 (7)	0.0009 (5)	0.0031 (5)	−0.0010 (5)
C8	0.0117 (7)	0.0158 (7)	0.0117 (6)	0.0026 (5)	0.0043 (5)	0.0009 (5)
C9	0.0161 (7)	0.0103 (7)	0.0153 (7)	−0.0009 (5)	0.0045 (5)	−0.0001 (5)
C10	0.0126 (7)	0.0166 (7)	0.0148 (7)	−0.0013 (6)	0.0041 (5)	0.0000 (6)
C11	0.0151 (7)	0.0163 (7)	0.0120 (7)	0.0022 (6)	0.0032 (5)	−0.0011 (5)
C12	0.0187 (7)	0.0123 (7)	0.0156 (7)	0.0016 (6)	0.0047 (6)	0.0005 (5)
C13	0.0151 (7)	0.0152 (7)	0.0151 (7)	−0.0026 (6)	0.0045 (5)	0.0004 (5)
C14	0.0161 (7)	0.0204 (8)	0.0164 (7)	0.0026 (6)	0.0038 (6)	−0.0037 (6)
C15	0.0162 (7)	0.0152 (8)	0.0263 (8)	−0.0004 (6)	0.0078 (6)	0.0022 (6)

### Geometric parameters (Å, º)

**Table:**

O1—C7	1.2034 (17)	C4—H4	0.9500
O2—C7	1.3717 (17)	C5—C6	1.408 (2)
O2—C8	1.3923 (16)	C6—C15	1.503 (2)
O3—N1	1.2333 (16)	C8—C13	1.389 (2)
O4—N1	1.2269 (16)	C8—C9	1.391 (2)
O5—C14	1.2123 (19)	C9—C10	1.387 (2)
O6—N2	1.2276 (17)	C10—C11	1.391 (2)
O7—N2	1.2305 (16)	C10—H10	0.9500
N1—C9	1.4615 (19)	C11—C12	1.395 (2)
N2—C5	1.4745 (19)	C11—C14	1.486 (2)
C1—C2	1.398 (2)	C12—C13	1.381 (2)
C1—C6	1.405 (2)	C12—H12	0.9500
C1—C7	1.4913 (19)	C13—H13	0.9500
C2—C3	1.385 (2)	C14—H14	0.992 (17)
C2—H2	0.9500	C15—H15A	0.9800
C3—C4	1.386 (2)	C15—H15B	0.9800
C3—H3	0.9500	C15—H15C	0.9800
C4—C5	1.380 (2)		
			
C7—O2—C8	118.77 (11)	C13—C8—C9	119.32 (13)
O4—N1—O3	124.66 (13)	C13—C8—O2	115.89 (13)
O4—N1—C9	117.76 (13)	C9—C8—O2	124.79 (13)
O3—N1—C9	117.57 (12)	C10—C9—C8	121.03 (13)
O6—N2—O7	123.78 (13)	C10—C9—N1	117.17 (13)
O6—N2—C5	119.28 (12)	C8—C9—N1	121.78 (13)
O7—N2—C5	116.88 (13)	C9—C10—C11	119.15 (14)
C2—C1—C6	122.21 (13)	C9—C10—H10	120.4
C2—C1—C7	116.91 (13)	C11—C10—H10	120.4
C6—C1—C7	120.81 (13)	C10—C11—C12	119.93 (14)
C3—C2—C1	120.48 (14)	C10—C11—C14	118.67 (14)
C3—C2—H2	119.8	C12—C11—C14	121.39 (14)
C1—C2—H2	119.8	C13—C12—C11	120.34 (14)
C2—C3—C4	119.32 (14)	C13—C12—H12	119.8
C2—C3—H3	120.3	C11—C12—H12	119.8
C4—C3—H3	120.3	C12—C13—C8	120.07 (14)
C5—C4—C3	118.99 (14)	C12—C13—H13	120.0
C5—C4—H4	120.5	C8—C13—H13	120.0
C3—C4—H4	120.5	O5—C14—C11	123.16 (15)
C4—C5—C6	124.48 (14)	O5—C14—H14	121.8 (10)
C4—C5—N2	115.48 (13)	C11—C14—H14	115.0 (10)
C6—C5—N2	120.04 (13)	C6—C15—H15A	109.5
C1—C6—C5	114.42 (13)	C6—C15—H15B	109.5
C1—C6—C15	122.28 (13)	H15A—C15—H15B	109.5
C5—C6—C15	123.30 (13)	C6—C15—H15C	109.5
O1—C7—O2	123.29 (13)	H15A—C15—H15C	109.5
O1—C7—C1	126.50 (13)	H15B—C15—H15C	109.5
O2—C7—C1	110.16 (12)		
			
C6—C1—C2—C3	−2.7 (2)	C6—C1—C7—O2	−131.49 (13)
C7—C1—C2—C3	174.41 (13)	C7—O2—C8—C13	128.15 (13)
C1—C2—C3—C4	1.6 (2)	C7—O2—C8—C9	−52.74 (18)
C2—C3—C4—C5	1.0 (2)	C13—C8—C9—C10	−3.7 (2)
C3—C4—C5—C6	−2.8 (2)	O2—C8—C9—C10	177.22 (12)
C3—C4—C5—N2	177.60 (13)	C13—C8—C9—N1	174.58 (13)
O6—N2—C5—C4	−139.54 (14)	O2—C8—C9—N1	−4.5 (2)
O7—N2—C5—C4	37.86 (17)	O4—N1—C9—C10	−37.16 (19)
O6—N2—C5—C6	40.80 (19)	O3—N1—C9—C10	141.65 (13)
O7—N2—C5—C6	−141.79 (14)	O4—N1—C9—C8	144.49 (14)
C2—C1—C6—C5	1.0 (2)	O3—N1—C9—C8	−36.7 (2)
C7—C1—C6—C5	−175.99 (12)	C8—C9—C10—C11	4.4 (2)
C2—C1—C6—C15	−178.84 (13)	N1—C9—C10—C11	−173.94 (13)
C7—C1—C6—C15	4.2 (2)	C9—C10—C11—C12	−1.8 (2)
C4—C5—C6—C1	1.7 (2)	C9—C10—C11—C14	177.84 (13)
N2—C5—C6—C1	−178.63 (12)	C10—C11—C12—C13	−1.5 (2)
C4—C5—C6—C15	−178.42 (14)	C14—C11—C12—C13	178.86 (13)
N2—C5—C6—C15	1.2 (2)	C11—C12—C13—C8	2.3 (2)
C8—O2—C7—O1	7.4 (2)	C9—C8—C13—C12	0.3 (2)
C8—O2—C7—C1	−170.43 (12)	O2—C8—C13—C12	179.48 (12)
C2—C1—C7—O1	−126.35 (16)	C10—C11—C14—O5	−166.20 (14)
C6—C1—C7—O1	50.8 (2)	C12—C11—C14—O5	13.4 (2)
C2—C1—C7—O2	51.37 (16)		

### Hydrogen-bond geometry (Å, º)

**Table:**

*D*—H···*A*	*D*—H	H···*A*	*D*···*A*	*D*—H···*A*
C10—H10···O5^i^	0.95	2.48	3.3457 (18)	152
C12—H12···O4^ii^	0.95	2.71	3.5321 (19)	145

Symmetry codes: (i) −*x*−1, *y*−1/2, −*z*+3/2; (ii) *x*, *y*+1, *z*.
